# A metabolite attenuates neuroinflammation, synaptic loss and cognitive deficits induced by chronic infection of *Toxoplasma gondii*

**DOI:** 10.3389/fimmu.2022.1043572

**Published:** 2022-12-22

**Authors:** Yan He, Daxiang Xu, Ziyi Yan, Yongshuai Wu, Yongsheng Zhang, Xiaokang Tian, Jinhang Zhu, Zhuanzhuan Liu, Wanpeng Cheng, Kuiyang Zheng, Xiaoying Yang, Yinghua Yu, Wei Pan

**Affiliations:** ^1^ Jiangsu Key Laboratory of Immunity and Metabolism, Department of Pathogen Biology and Immunology, Xuzhou Medical University, Xuzhou, Jiangsu, China; ^2^ The First Clinical Medical College, Xuzhou Medical University, Xuzhou, Jiangsu, China; ^3^ National Experimental Teaching Demonstration Center of Basic Medicine (Xuzhou Medical University), Xuzhou, Jiangsu, China; ^4^ The Second Clinical Medical College, Xuzhou Medical University, Xuzhou, Jiangsu, China

**Keywords:** *Toxoplasma gondii*, Acod1/itaconate axis, neuroinflammation, cognition, synaptic plasticity, metabolic reprogramming, hippocampus

## Abstract

**Background:**

Neurodegenerative diseases including AD is currently one of intractable problems globally due to the insufficiency of intervention strategies. Long-term infection of Toxoplasma gondii (T. gondii) can induce cognitive impairment in hosts, which is closely implicated in the pathogenesis of neurodegenerative diseases. Aconitate decarboxylase 1 (Acod1) and its produced metabolite itaconate (termed Acod1/itaconate axis), have recently attracted extensive interests due to its anti-inflammatory role in macrophages. However, whether the axis can influence cognitive function remains unknown.

**Methods:**

A chronic T. gondii-infected mice (C57BL/6J) model was established via administration of cysts by gavage. Novel location (NL), novel object recognition (NOR), Y-maze spatial memory and nest building tests were used to evaluate the behavior performance. Transmission electron microscopy, immunofluorescence, RT-PCR, western-blotting and RNA sequencing were utilized to determine the pathological changes, neuroinflammation and transcription profile in hippocampus tissues post infection, respectively. Moreover, the protective effect of Acod1/itaconate axis in T. gondii-induced cognitive deficits was evaluated.

**Results:**

We found that the latent infection of the parasite impaired the cognitive function, which was assessed behaviorally by novel location (NL), novel object recognition (NOR), Y-maze spatial memory and nest building tests. RNA sequencing of hippocampus showed that the infection downregulated the expression of genes related to synaptic plasticity, transmission and cognitive behavior. To our attention, the infection robustly upregulated the expression of genes associated with pro-inflammatory responses, which was characterized by microglia activation and disorder of Acod1/itaconate axis. Interestingly, administration of dimethyl itaconate (DI, an itaconate derivative with cell membrane permeability) could significantly ameliorate the cognitive deficits induced by T. gondii, which was proved by improvement of behavior performance and synaptic ultrastructure impairment, and lower accumulation of pro-inflammatory microglia. Notably, DI administration had a potential therapeutic effect on the cognitive deficits and synaptic impairment induced by the parasitic infection.

**Conclusions:**

Overall, these findings provide a novel insight for the pathogenesis of T. gondii-related cognitive deficits in hosts, and also provide a novel clue for the potential therapeutic strategies.

## 1 Introduction

Neurodegenerative diseases, including Alzheimer’s disease (AD) and related dementias, represent a major contributor to morbidity, drastically impaired quality of life, and health care costs in an increasingly aging population (1). Frustratingly, the diseases currently lack effective therapies (2). *Toxoplasma gondii*, a neurotropic parasite, has a wide host range, and approximately infects one-third of the world’s population (3). There is accumulating evidence suggests that the parasite can act as a vital factor for neurodegenerative diseases. It’s reported that in human, the higher anti-*T. gondii* antibody is closely associated with the lower cognitive function, characterized by working memory impairment and worse executive ability (4, 5). Moreover, there is a positive correlation between *T. gondii* infection and the incidence of various psychiatric and neurological disorders including AD (6–8). In support, *T. gondii* infection is reported to impair cognitive function in murine models (9, 10). Notably, a recent study has demonstrated that the chronic infection of the parasite can directly induce AD-like behavior performance, including impaired spatial memory and alternations in social novelty recognition (11).

The hippocampus, lying just beneath the neocortex, is implicated in cognitive processing, social recognition and memory (12–14). In most neurodegenerative disorders, the reduced synaptic density and synaptic plasticity in the hippocampus has been closely correlated with cognitive impairment (15–17). Interestingly, there is a disperse distribution of cysts in the brain of *T. gondii* infected mice, including hippocampus (18). Notably, a recent study reports that *T. gondii* infection can downregulate the expression of proteins maintaining synaptic structure, functional integrity and synaptic plasticity (synaptophysin and PSD95) in the hippocampus of mice (9). Thus, the compromised hippocampus is a key brain area related with the cognitive decline induced by *T. gondii* infection.

Neuroinflammation plays a vital role in the progression of neurodegenerative diseases (19). In AD, long-term activation of microglia and astrocytes (20), can release extensive pro-inflammatory cytokines including IL-1β, IL-6, and TNF-α (21). It is reported that these cytokines can directly induce neuronal apoptosis and synaptic dysfunction (22). Moreover, activated microglia can engulf synapses and destroy synaptic ultrastructure (23). They jointly result in the cognitive impairment (24). Interestingly, recent studies have also indicated that neuroinflammation induced by *T. gondii* is closely associated with the alteration of behavior performance (25–29). Concurrently, neuronal changes including reduction of dendritic spine length and density (30, 31), have been reported in the mice post *T. gondii* chronic infection. It is therefore proposed that alleviating neuroinflammation may improve the parasite-induced cognitive decline *via* optimizing synaptic ultrastructure and plasticity.

Immunometabolism, the newly emerging discipline, uncovers that the metabolites or metabolic events can precisely shape the function and fate of immune cells (32). This opens a new direction for improving cognition by manipulating neuroinflammation (33). Aconitate decarboxylase 1 (Acod1), also known as immune responsive gene 1 (IRG1), is a key enzyme in tricarboxylic acid cycle (TCA cycle), which catalyzes cis-aconitate to produce itaconate during inflammation (34, 35). Interestingly, the Acod1/itaconate axis has been demonstrated to be a key node that links immunity and metabolism in macrophages (35). Notably, recent studies have revealed that itaconate can master inflammatory response in macrophages *via* reprogramming metabolic flux (34, 36–38). Moreover, the derivatives of itaconate, such as Dimethyl itaconate (DI), have attracted extensive interests in the treatment of inflammatory diseases (36, 39–45). In addition, recent studies showed that DI can ameliorate neuroinflammation in a mouse model of multiple sclerosis (46), and can attenuate memory impairment in the mice model of AD (47). Here we were interested in whether the Acod1/itaconate axis has a protective effect in *T. gondii*-induced cognitive decline.

In the present study, we reported that chronic infection of TgCtwh6, a unique *T. gondii* strain prevalent in China (48–50), can induce cognitive deficits in mice, which is accompanied by neuroinflammation, impairment of ultrastructure and plasticity of synapses. Moreover, RNA sequencing (RNA-seq) revealed that in the hippocampus, TgCtwh6 infection significantly suppressed the pathways associated with cognition, behavior and synaptic plasticity, while activating extensive inflammatory pathways. Correspondingly, the infection induced complex metabolic reprogramming in the hippocampus. To our attention, the disorder of Acod1/itaconate axis was identified post infection. Importantly, we demonstrated for the first time that supplementation of DI, a cell-permeable itaconate derivative, can effectively attenuate TgCtwh6-induced cognitive impairment. Taken together, these findings provide novel information for the pathogenesis and treatment of *T. gondii*-related neurodegenerative diseases.

## 2 Materials and methods

### 2.1 Animals, parasite

C57BL/6J mice (7 weeks old) were obtained from Jiangsu Jicui Pharmaceutical Technology Corporation (Jiangsu Province, China), and housed in environmentally controlled conditions (temperature 24°C, 12 h light/dark cycle) and given free access to standard food and water in specific pathogen free (SPF) experimental animal Center of Xuzhou Medical University. The mice were acclimatized for 1 week before the experiment. TgCtWh6, a strain of *T. gondii* that often causes chronic infection and prevails in China (48–50) was gifted by the laboratory of Professor Jilong Shen. The cysts of TgCtWh6, were isolated from the brains of infected mice to establish the *T. gondii* infected model in mice.

### 2.2 Model establishment of *Toxoplasma gondii* chronic infection induced cognitive impairment

Mice were randomly divided into two groups: (I) Mice received the phosphate buffer saline (PBS) by gavage as a control (Con) group; (II) Mice received the cysts of TgCtWh6 by gavage (10 cysts for each mouse) as Tg group. The exact process of infection was carried out as previously described (51). After 4 weeks post infection, the cognitive behavior tests were performed. Mice were sacrificed 4 days after behavior testing with CO_2_. The hippocampus tissues and other brain tissues were collected for further analyses.

### 2.3 The prophylactic effect of DI on *Toxoplasma gondii*-induced cognitive impairment

The strategy of the experiment was shown in **Figure 4A**. Briefly, mice were randomly divided into four groups. Con and Tg groups were performed as mentioned in **2.2**. In Con+Veh group, mice received PBS as vehicle control. In Con+DI group, control mice received 40 mg DI (Cat. 617527, Sigma-Aldrich, St. Louis, USA) per kilogram body weight. In Tg+Veh group, the Tg infected mice received PBS. In Tg+DI group, the Tg infected mice received the same dose of DI. DI administration (intraperitoneal injection, twice per week), started at one week before *T. gondii* infection until the ending of behavioral tests. The hippocampus tissues were collected for further analyses.

### 2.4 The therapeutic effect of DI on *Toxoplasma gondii*-induced cognitive impairment

The strategy of the experiment was shown in **Figure 7A**. In brief, mice were infected with the cysts of TgCtWh6 by gavage. After four weeks post infection, these mice were divided into two groups: mice intraperitoneally administrated with 40 mg DI per kilogram body weight as Tg+DI treatment group, while Tg mice received PBS as Tg+Veh group. DI administration (twice per week) lasted 3 weeks until the ending of behavioral tests. The hippocampus tissues were collected for further analyses.

### 2.5 Behavioral tests

The novel location (NL), novel object recognition (NOR), Y-maze spatial memory and nest building tests were carried out to evaluate the effects of *T. gondii* infection and DI supplementation on spatial memory, recognition memory and flexibility in fine motion of mice.

The NL test was performed as previously described (52). Briefly, there are three stages in the NL. The first stage is habituation, in which a mouse is allowed to explore the open field for 5 min. After 24 h, beginning the training stage, in which the mouse allowed to explore the arena for 5 min with 2 identical objects placed parallel. After 1 h, the retention session takes place. One of the objects is shifted to a new diagonal position, and mice are allowed to explore the arena for 5 min. The discrimination index was evaluated by using the formula: [Time spent with the object moved to a novel place/Total time spent in exploring both the object moved to a novel place and the object remaining in the familiar place] × 100%. All experimental arenas were wiped clean with 75% ethanol solution and ddH_2_O after each trial.

The NOR test was performed as previously described (53). Briefly, there are three stages in the NOR test. The first stage is habituation, in which a mouse is allowed to explore the open field for 5 min. After 24 h, beginning the training stage, in which the mouse allowed to explore the arena for 5 min with 2 identical objects placed parallel. After 1 h, retention session takes place. Mice are allowed to explore the arena with one of the familiar objects and one novel object placed parallel for 5 min. The discrimination index was evaluated by using the formula: [Time spent with novel object/(Time spent with old object + Time spent with novel object)] × 100%. All experimental arenas were wiped clean with 75% ethanol solution and ddH_2_O after each trial.

The Y-maze spatial memory test was performed as previously described (54). The Y-maze, which relies on the animals’ endogenous drive for exploring spatial novelty (55), consists of three similar arms (30 cm × 10 cm × 16 cm), and at an angle of 120° to each other. Animals are placed in the start arm facing away from the center of the maze and facing toward the center. There are two stages in the Y-maze. The first stage is the phase of acquisition, in which one arm entrance of the Y-maze was blocked at random, and the experimental mice was randomly placed in one of the two other arms, with its head pointing away from the center of the maze. The mice are allowed to visit the two accessible arms of the maze for 5 minutes. The second stage is the phase of retrieval, the mice was allowed to explore all three arms for 5 min. The number of visits to each arm was recorded for each trial. The mice with memory impairment spend less time exploring in their new arms. The ratio of time spent in novel arm (%) was calculated as: [Number of times spent in novel arms/Total number of times spent in all arms]×100%. All experimental arenas were wiped clean with 75% ethanol solution and ddH_2_O after each trial.

The nest building test was performed as previously described (56), which aims to evaluate spontaneous rodent behavior. Briefly, transfer the mice to individual testing cages with free access to standard food and water. Approximately 1 h before the dark phase, and place one nestlet weighing 3 g in each cage, in the case of no other environmental enrichment items. Assess the deacon nest score and untorn nestlet weight next morning to evaluate the activities of daily living typically altered in patients with cognitive impairment. According to a previously described scoring system, assess the nest scores on a definitive 5-point nest-rating scale (56).

### 2.6 Transmission electron microscope

After transcardial perfusion with saline, brain tissues were taken out freshly and 1 mm^3^ of tissue blocks from the cornus ammonis (CA1) region of hippocampus. Samples were rapidly fixed in a 2% paraformaldehyde-2.5% glutaraldehyde mixture for 24 h. The hippocampal tissues were quickly dissected and separated into thin slices after fixation. They were fixed immediately with 2.5% glutaraldehyde at 4°C overnight. After washing 3 times in PBS, these slices were fixed in 1% osmium tetroxide, stained with 2% aqueous solution of uranyl acetate, and then dehydrated with different concentrations of ethanol (30% ~ 100%) and acetone gradient. Finally, they were embedded in epoxy resin. Ultra-thin sections (70 nm) were cut with ultramicrotome, collected on copper grids, and then stained with 4% uranyl acetate and 0.5% lead citrate. Synapses are classified into asymmetric and symmetric synapses, or Gray I type and Gray II type synapses, which are considered to mediate excitatory and inhibitory transmission, respectively. Asymmetric synapses have prominent postsynaptic densities and relatively wide synaptic clefts while symmetric synapses are with pre- and postsynaptic densities of equal thickness and narrower synaptic clefts. In the present study, asymmetric synapses were examined for excitatory synaptic measurement. The PSD thickness was evaluated as the length of a perpendicular line traced from the postsynaptic membrane to the most convex part of the synaptic complex. The widths of the synaptic clefts (SCs) were the average of the widest and narrowest portions of the synapse. The synaptic curvature was calculated by the arc length of the presynaptic membrane divided by chord length. In the present, we analyzed four indexes (Postsynaptic density, synaptic clefts width and the curvature of the synaptic interface) by using Image J software (Version 1.53n, https://imagej,nih.gov/ij/) (57).

### 2.7 Immunofluorescence

The immunohistochemical staining has been described in our previous study (58). Briefly, fixed tissues were embedded in paraffin and sectioned at 20 μm, using PBS washed 3 times for 10 min, and then washed in 1% H2O2 in PBS for 30 min. All sections were blocked with 5% normal goat serum and incubated with indicated primary antibodies at 4°C overnight. Primary antibodies were rabbit anti-calcium-binding adapter molecule 1 (Iba1, Ab178847, Abcam, 1:100 dilution). Following primary antibody incubation, sections were washed with PBS and then incubated with goat anti-rabbit IgG H&L (ab6702, Abcam, 1: 500 dilution) at 37°C for 2 h. Finally, using the DAB peroxidase substrate kit (Cell Signaling Technology, Boston, USA) to wash the sections and the sections were counterstained with hematoxylin (Sigma-Aldrich, St. Louis, USA). Six fields from three sections of each mouse were viewed by OLYMPUS IX51 microscope (Tokyo, Japan) and digital photographs were captured. Image J software was used to quantify Iba1 immunoreactivity on each field.

### 2.8 RNA extraction and quantitative RT-PCR

Total RNA was extracted from the hippocampus tissues homogenized in RNA isolater (Vazyme Biotech Co.,Ltd, Nanjing, China). Using a spectrophotometer (DU800, Beckman Coulter Inc., Brea, CA, USA), the total RNA concentration was confirmed at 260 nm and 280 nm. Then, 1 μg of purified RNA was reverse-transcripted for RT-PCR to generate cDNA with HiScript II Q RT SuperMix for qPCR (+g DNA wiper) (Vazyme Biotech Co.,Ltd, Nanjing, China). qPCR was performed using the ChamQ SYBR qPCR Master Mix (Vazyme Biotech Co.,Ltd, Nanjing, China) and determined on a real-time PCR detection system (Roche, Switzerland). The relative mRNA expression level was determined with the 2-ΔΔCt method with β-actin as the internal reference control. Primer sequences were shown in **Supplementary Table 1**.

### 2.9 Western blotting

The hippocampus of mice were homogenized in ice-cold RIPA lysis buffer, supplemented with complete EDTA-free protease inhibitor cocktail and PhosSTOP Phosphatase Inhibitor. Then, the homogenate was sonicated six times for 4 s, at 6 s intervals on ice, and centrifuged at 12,000 g for 20 min at 4°C. The supernatants were collected, and the protein concentration was quantitated by BCA assay. Equal amounts of protein were separated by sodium dodecyl sulfate-polyacrylamide gel electrophoresis (SDS-PAGE) and transferred onto polyvinylidene difluoride (PVDF) membranes. The membrane was blocked with 5% non-fat milk at room temperature for 1 h, and then incubated with the primary antibody at 4°C overnight. The primary antibodies included: Acod1 (Cell Signaling, 17805S, 1: 1000 dilution), Sdha (Cell Signaling, 11998S, 1: 1000 dilution), Pkm2 (Wanleibio, WL03290, 1: 1200 dilution) and β-actin (ABclonal, AS003, 1:7000 dilution). Following 3 washes in TBST, the membrane was incubated with HRP-inked anti-rabbit IgG secondary antibody (ABclonal, AS014, 1:5000 dilution) at room temperature for 1 h. After washing 3 times with TBST, the protein bands were detected with Clarity™ ECL western blot substrate (Bio-Rad, 1,705,060) and visualized using the ChemiDoc Touch imaging system (Bio-Rad).

### 2.10 RNA sequencing

Fresh hippocampus tissues of mice were collected to analyze the whole profile of transcriptome. According to the manufacturer’s protocol, we use trizol reagent kit (Invitrogen, Carlsbad, CA, USA) to extracte total RNA from the tissue. After total RNA was extracted, eukaryotic mRNA was enriched by Oligo(dT) beads, while prokaryotic mRNA was enriched by removing rRNA by Ribo-ZeroTM Magnetic Kit (Epicentre, Madison, WI, USA). Then the enriched mRNA was fragmented into short fragments by using fragmentation buffer and reversed transcripted into cDNA with random primers. Second-strand cDNA were synthesized by DNA polymerase I, RNase H, dNTP and buffer. Then the cDNA fragments were purified with QiaQuick PCR extraction kit (Qiagen, Venlo, The Netherlands), end repaired, poly(A) added, and ligated to Illumina sequencing adapters. The ligation products were size selected by agarose gel electrophoresis, PCR amplified, and sequenced using Illumina HiSeq2500 by Gene Denovo Biotechnology Co. (Guangzhou, China). DEGs (differentially expressed genes) were assessed by analysis of differential RNA expression between two groups. Differential gene screening criteria were the parameter of false discovery rate (FDR) below 0.05 and an absolute fold change of 2 or greater. DEGs were visualized using volcano maps.

Bioinformation enrichment analysis includes GO enrichment analysis and KEGG enrichment analysis. We used the DAVID Bioinformatics Resources 6.8 (https://david.ncifcrf.gov/), an analysis of the enrichment of biological information analysis of differentially expressed genes. GO analysis includes cellular component (CC), which is used to describe the subcellular structure, location and macromolecular complexes, such as nucleoli and telomeres; molecular function (MF), used to describe the function of genes, gene products, such as carbohydrate binding or ATP hydrolase activity; biological process (BP), referring to the ordered combination of molecular functions to achieve a wider range of biological functions, such as mitosis or purine metabolism. KEGG pathway enrichment analysis was performed by using the database (http://www.kegg.jp/). *P* value of 0.05 or less was considered as a threshold. Pathways meeting this condition were defined as significantly enriched pathways in DEGs.

For gene set enrichment analysis (GSEA), in order to identify whether a set of genes in specific GO terms or pathways terms shows significant differences in two groups, we performed gene set enrichment analysis using software GSEA (59) and MSigDB (59). Briefly, we input gene expression matrix and rank genes by SinaltoNoise normalization method. Enrichment scores and *P* value was calculated in default parameters. Gene sets with the parameter of normalized enrichment score (NES) ≥1, *P* value < 0.05 and false discovery rate (FDR) <0.05 were considered significantly enriched.

### 2.11 Statistical analysis

Data were presented as mean ± standard error of the mean (SEM) and analyzed using GraphPad Prism software 8.0. After data were performed the Shapiro-Wilk normality test, the Student’s t test was used to compare two groups, while One-Way analysis of variance (ANOVA) was used for the comparisons of four groups, followed by the *post hoc* Tukey-Kramer test for multiple comparisons. A *P* value < 0.05 was considered to indicate statistical significance.

## 3 Results

### 3.1 *Toxoplasma gondii* chronic infection impairs cognitive function in mice

**Figure 1:**
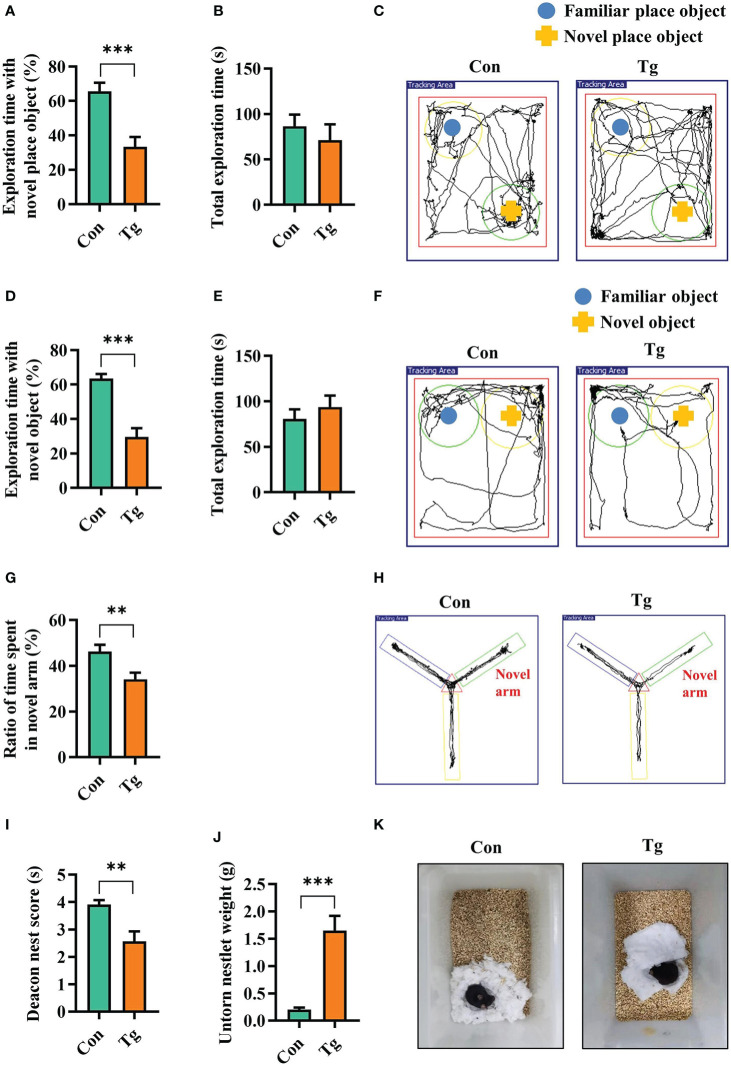
*T. gondii* chronic infection impairs cognitive function in mice. **(A)** Percentage of time spent with the object in the novel place to total object exploration time and **(B)** the total object exploration time in the novel location test were recorded. **(C)** Representative track plots of Con and Tg groups recorded by SMART video tracking system in the testing phase. **(D)** Percentage of time spent with the novel object to total object exploration time and **(E)** the total object exploration time in the novel object recognition test were recorded. **(F)** Representative track plots of Con and Tg groups recorded by SMART video tracking system. **(G)** Percentage of time spent with the novel arm to total arm exploration time in Y-maze spatial memory test. **(H)** Representative example of spatial memory in the Y-maze test recorded by SMART video tracking system. Note that the control mouse spent more time exploring the novel arm whereas the mice infected with *T. gondii* did not show preference to the novel arm. **(I)** The nest score and **(J)** untorn nestlet weight (amount of untorn nesting material) (*n* = 10 mice for each group). **(K)** Representative images of nesting result in Con and Tg groups. Con, control mice; Tg, *T. gondii* infected mice. Values are mean ± SEM. **P<0.01, ***P<0.001.

The cognitive function (including recognition memory, spatial memory and ability to perform activities of daily living), can be evaluated by novel location, novel object recognition tests, Y-maze spatial memory test, and nest building test, respectively (52–54, 56). We assessed whether *T. gondii* chronic infection could impair cognitive function in mice. In novel location test, the Tg mice exhibited a lower percentage of time spent with the object in a novel place than that in Con mice (*P* < 0.001, **Figures 1A, C**). In novel object recognition test, Tg mice spent less time with new object, and showed a lower percentage of time spent with new object (*P* < 0.001, **Figures 1D, F**), reflecting the worse object recognition memory. The difference of the above two behavior tests was not attributed to the variant general activity because the Con and Tg groups had a similar total exploration time and distance during the test phase (*P*> 0.05, **Figures 1B, E**; **Supplementary Figures 1A, B**). In Y-maze spatial memory test, Tg infection could greatly worsen spatial recognition memory with decreasing the novel place discrimination index and the percentage of time spent in novel arm compared with Con group (*P* < 0.01, **Figures 1G, H**). In nest building test, Tg group had lower deacon nest score than that of the Con mice (*P* < 0.01, **Figures 1I, K**). In contrast, the untore nestlet weight in Tg group was significantly increased than that in Con group (*P* < 0.001, **Figures 1J, K**), which implied that the parasitic infection can weaken the ability of daily living in mice. Overall, these data support that *T. gondii* chronic infection induces cognitive impairment in mice. Furthermore, the Tg mice showed lower body weight gain (*P* < 0.001, **Supplementary Figure 1C**) and increased piloerection (data not shown).

### 3.2 *Toxoplasma gondii* chronic infection disturbs the expression of genes related to cognition, synaptic plasticity and transmission in the hippocampus of mice

**Figure 2:**
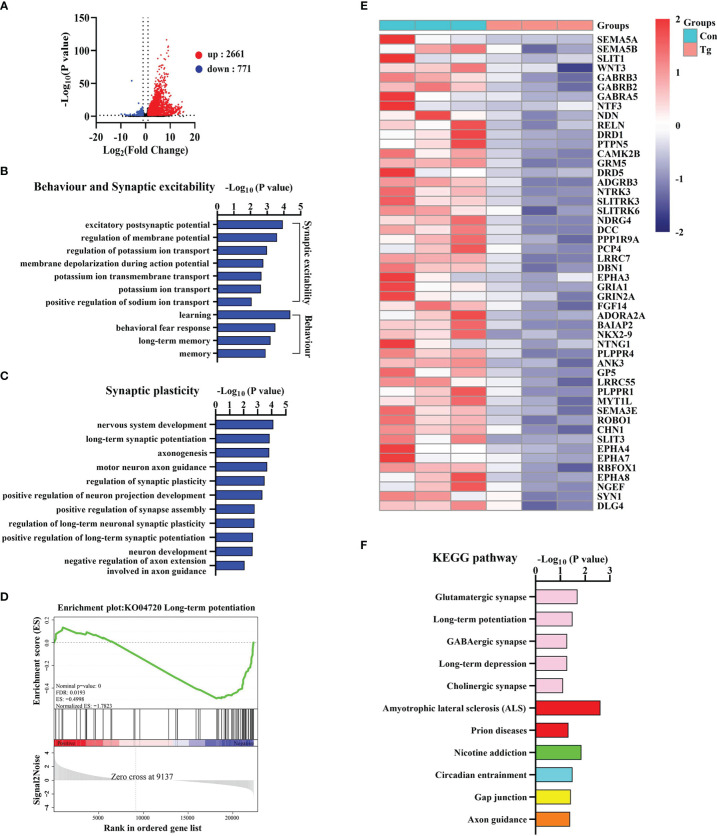
*T. gondii* chronic infection disturbs the expression of genes related to cognition, synaptic plasticity and transmission in the hippocampus of mice. **(A)** The volcano plot shows the distributions of differentially expressed genes (DEGs) between Con and Tg mice. **(B)** The biological processes associated with behavior and synaptic excitability are downregulated in *T. gondii* infected mice. **(C)** The biological processes associated with synaptic plasticity are downregulated in *T. gondii* infected mice. **(D)** GSEA analysis revealed that genes involved in long-term potentiation were significantly downregulated. **(E)** Heatmap demonstrating the relative expression of core genes in the enrichment plot (*n* = 3) are upregulated in Tg group in comparison to Con group. **(F)** The enriched KEGG pathways related to axon guidance and synapse related pathways in *T. gondii* infected mice. Columns with different colors represent different classification in level 2. Con, control mice; Tg, *T. gondii* infected mice.

To uncover how *T. gondii* chronic infection induces cognitive deficits, we characterized the transcriptomic profile in the hippocampus of mice post the parasitic infection. Differentially expressed genes (DEGs) were identified after filtering the raw data based on *P* < 0.05 and |fold changes| > 1.5. There were 3432 DEGs, including 2661 upregulated genes and 771 downregulated genes post chronic infection (**Figure 2A**). Gene ontology (GO) analysis was carried out to seek the significantly enriched terms in the downregulated DEGs caused by *T. gondii* infection. Interestingly, these significantly enriched terms were found to associate with learning, long-term memory, synaptic excitability, synaptic plasticity and synaptic transmission (**Figures 2B, C**; **Supplementary Figure 2C**). Moreover, the expression of the representative genes related to synaptic plasticity, was downregulated due to the chronic infection and listed in the heatmap (**Figure 2E**).

Furthermore, we observed 4 significantly enriched gene sets (NES ≤ 1, *P* < 0.05 and FDR < 0.25) including “Long-term potentiation”, “GABA-A receptor activity”, “Trans-synaptic signaling” and “Neurotransmitter receptor activity” (**Figure 2D**; **Supplementary Figures 2D–F**). In addition, KEGG analysis showed that the axon guidance and synapse related pathways (glutamatergic synapse, long-term potentiation, GABAergic synapse, *etc.*) were significantly enriched post *T. gondii* infection (**Figure 2F**). Collectively, these results indicated that long-term infection of *T. gondii* inhibits the expression profile of the genes associated with cognition, synaptic plasticity and transmission, which further explains the abnormal behavior performance induced by *T. gondii*.

### 3.3 *Toxoplasma gondii* chronic infection triggers extensive neuroinflammation along with the Acod1/itaconate axis disorder in the hippocampus of mice

**Figure 3:**
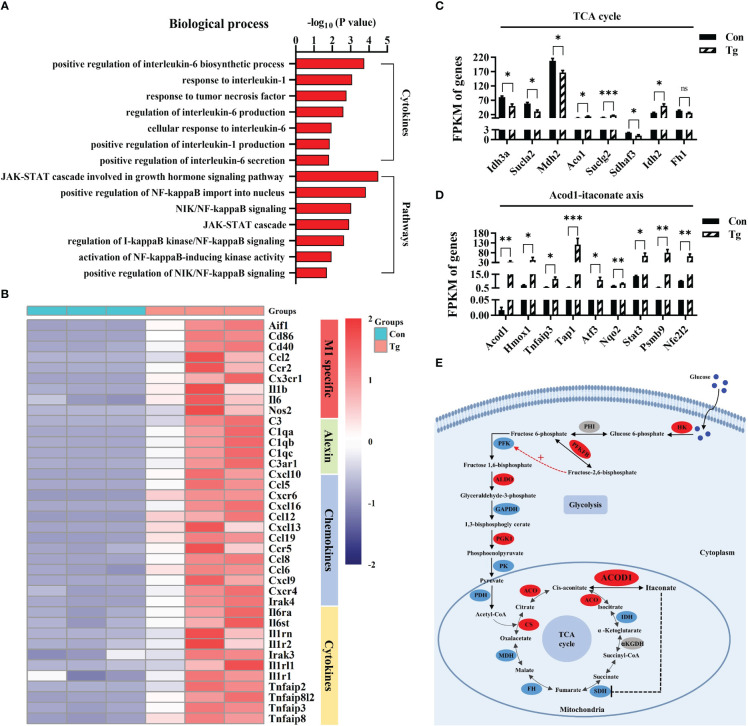
*T. gondii* chronic infection triggers extensive neuroinflammation along with disorder of the Acod1/itaconate axis in the hippocampus of mice. **(A)** The biological processes associated with cytokines and inflammatory pathways are upregulated in *T. gondii* infected mice. **(B)** Heatmap demonstrating the relative expression of core genes related to M1 specific, alexin, chemokines and cytokines in the enrichment plot (*n* = 3) are upregulated in Tg group in comparison to Con group. Normalized expression of selected genes regulating **(C)** TCA cycle and **(D)** Acod1-itaconate axis. **(E)** The overview of metabolic reprogramming in hippocampus in *T. gondii* infected mice. The metabolism diagram shows the expression profile of key enzymes. Glycolysis pathways were significantly upregulated while TCA cycle were dramatically broken. Enzymes identified in our study are marked in red oval (upregulated) and blue oval (downregulated), while other enzymes are marked in grey oval. Hk, hexokinase; Phi, phosphate isomerase; Pfkfb, fructose-2,6-biphosphatase; Pfk, phosphofructokinase; Aldo, aldolase; Gapdh, glyceraldehyde-3-phosphate dehydrogenase; Pgk1, phosphoglycerate kinase 1; Pk, pyruvate kinase; Pdh, pyruvate dehydrogenase complex; Cs, citrate synthase; Aco, aconitase; Acod1, aconitate decarboxylase 1; Idh, isocitrate dehydrogenase; αKGDH, α-ketoglutarate dehydrogenase; Sdh, succinate dehydrogenase; Fh, fumarate hydratase; Mdh, malate dehydrogenase. Con, control mice; Tg, *T. gondii* infected mice. Values are mean ± SEM. *P<0.05, **P<0.01, ***P<0.001.

Interestingly, we found that most upregulated DEGs in the infected hippocampus of mice were related to pro-inflammatory response. GO analysis showed that these DEGs were significantly enriched in the production of several pro-inflammatory cytokines (TNF-α, IL-6, and IL-1), and inflammation-related signaling pathways, such as NF-κB, JAK-STAT (**Figure 3A**). We noticed that specific markers of pro-inflammatory microglia including Aif1, Cd86, Cd40, Ccl2, Ccr2, Cx3cr1, IL-1β, IL-6, and Nos2 (60, 61) were significantly upregulated post infection (**Figure 3B**). Furthermore, we verified the mRNA expression alteration of Cd86 and Cd11c (markers of pro-inflammatory microglia) in the hippocampus of mice (*P*< 0.01) (**Supplementary Figures 3A, B**). These results indicated that *T. gondii* chronic infection induces extensive neuroinflammation, characterized by microglial activation.

Metabolic events can precisely determine the function and fate of immune cells (32). We further characterized the metabolic profile of hippocampus post *T. gondii* infection. It is most likely that the infection hampered the tricarboxylic acid (TCA) cycle (**Figure 3C**), because several genes (such as Sdhaf3, Sucla2, Idh3a and Mdh2) encoded the key enzymes in TCA cycle were significantly downregulated. To our attention, Acod1/itaconate axis in TCA cycle, a key node that links metabolism and immunity in macrophages (35), was disordered post infection (**Figures 3D, E**). Acod1 can catalyze cis-aconitate to produce itaconate during inflammation (34, 35). We found that the infection significantly upregulated the expression of Acod1 and its downstream genes (Atf3, Stat3, Nfe2l2 and Nrf2, *etc.*) (39, 62, 63) (**Figure 3D**). We confirmed that the mRNA expression of Mdh2 and Idh2 was altered post infection (*P*< 0.01, **Supplementary Figures 3C, D**). Moreover, we observed that the infection upregulated the protein expression of Acod1 while downregulating Sdha protein expression (*P*< 0.05, **Supplementary Figures 3E, F**). Furthermore, the mRNA expression of several genes related with glycolysis and fatty acid β-oxidation was upregulated due to the infection (*P*< 0.05, **Supplementary Figures 3G, H**). We verified the elevated protein expression of Pkm2, a key enzyme involved in glycolysis post infection (*P*< 0.01, **Supplementary Figure 3I**). Taken together, these results indicate that *T. gondii* chronic infection induces metabolic reprogramming in the hippocampus of mice, which is closely associated the neuroinflammation.

### 3.4 Dimethyl itaconate ameliorates the neuroinflammation in *Toxoplasma gondii* infected mice

**Figure 4:**
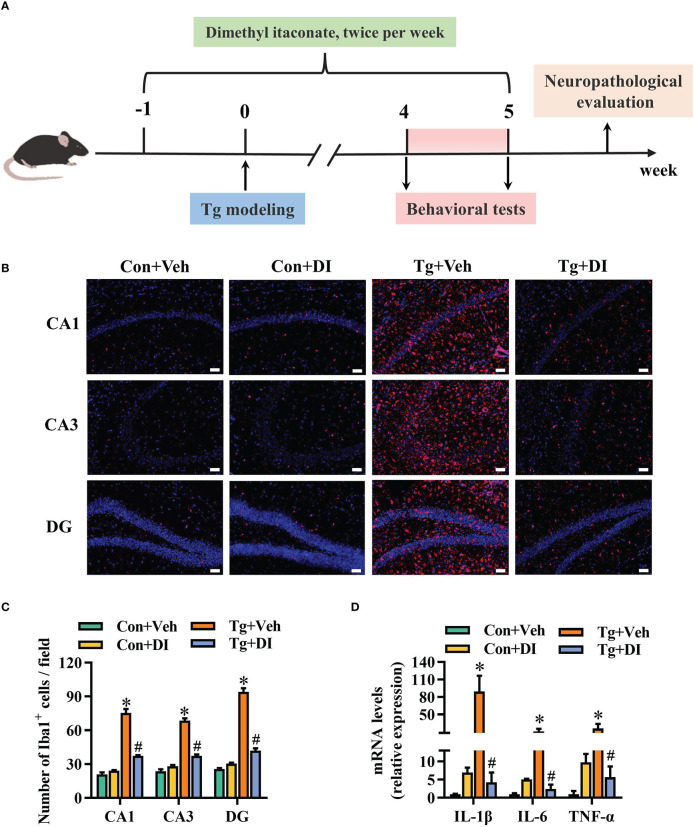
Dimethyl itaconate ameliorates the neuroinflammation in *T. gondii* infected mice. **(A)** Schematic timeline for DI treatment on cognitive deficits induced by *T. gondii* infection in mice. **(B)** Representative immunofluorescent staining of the Iba-1^+^ cells in CA1, CA3 and DG of the hippocampus (*n* = 3). **(C)** The quantification of Iba-1^+^ cells number in CA1, CA3 and DG of the hippocampus (*n* = 3, 10 images per mouse per region, scale bar: 50 μm). **(D)** The mRNA expression of IL-1β, IL-6 and TNF-α in the hippocampus (*n* = 3). Con+Veh, control mice with vehicle control treatment; Con+DI, control mice with DI treatment; Tg+Veh, *T. gondii* infected mice with vehicle control treatment; Tg+DI: *T. gondii* infected mice with DI treatment. Values are mean ± SEM. *P<0.05 vs. control (Con+Veh). ^#^ *P* < 0.05 vs. *T. gondii* infected (Tg+Veh).

The studies on *in vitro* anti-inflammatory effect of itaconate have recently attracted extensive interests (34, 36, 39, 64). Dimethyl itaconate (DI), an itaconate derivative with membrane permeability, has shown a potential in preventing inflammatory diseases including neuroinflammation (46). Here, we investigated whether DI could improve the neuroinflammation triggered by *T. gondii* infection. The strategy was shown in **Figure 4A**. In brief, DI administration (twice per week), started at one week before *T. gondii* infection until the ending of behavioral tests. Using Iba-1 as the activation marker of microglia, we observed the increased microglia number in the hippocampal CA1, CA3 and DG regions of the Tg mice (*P* < 0.05, **Figures 4B, C**). However, DI supplementation significantly reduced the microglia number in these areas (*P* < 0.05, **Figures 4B, C**). Moreover, the mRNA levels of pro-inflammatory cytokines (IL-1β, IL-6, TNF-α) were significantly higher in the hippocampus of Tg+Veh group than in the Con+Veh and Tg+DI groups (*P* < 0.05, **Figure 4D**). These results showed that DI attenuates the neuroinflammation induced by *T. gondii* infection.

### 3.5 Dimethyl itaconate improves the cognitive deficits in *Toxoplasma gondii* infected mice

**Figure 5:**
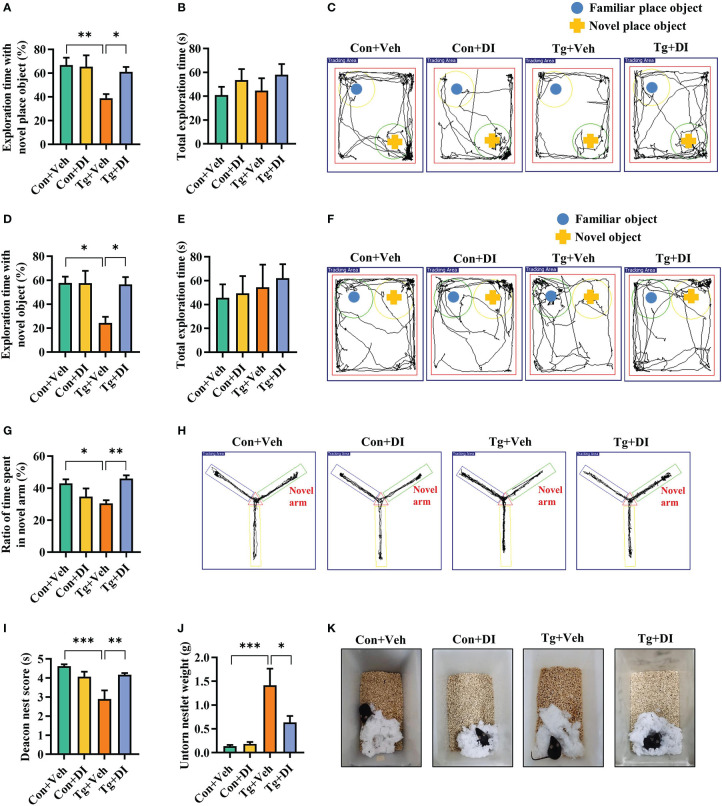
Dimethyl itaconate improves the cognitive deficits in *T. gondii* infected mice. **(A)** Percentage of time spent with the object in the novel place to total object exploration time and **(B)** the total object exploration time in the novel location test were recorded. **(C)** Representative track plots of Con+Veh, Con+DI, Tg+Veh and Tg+DI groups recorded by SMART video tracking system in the testing phase. **(D)** Percentage of time spent with the novel object to total object exploration time and **(E)** the total object exploration time in the novel object recognition test were recorded. **(F)** Representative track plots of Con+Veh, Con+DI, Tg+Veh and Tg+DI groups recorded by SMART video tracking system in the testing phase. **(G)** Percentage of time spent with the novel arm to total arm exploration time in Y-maze spatial memory test. **(H)** Representative example of spatial memory in the Y-maze test recorded by SMART video tracking system. Note that the mice in Tg+DI group spent more time exploring the novel arm than the mice infected with *T. gondii.* **(I)** The nest score and **(J)** untorn nestlet weight (amount of untorn nesting material) (*n* = 10 mice for each group). **(K)** Representative nesting result of Con+Veh, Con+DI, Tg+Veh and Tg+DI groups. Con+Veh, control mice with vehicle control treatment; Con+DI, control mice with DI treatment; Tg+Veh, *T. gondii* infected mice with vehicle control treatment; Tg+DI: *T. gondii* infected mice with DI treatment. Values are mean ± SEM. *P<0.05, **P<0.01, ***P<0.001.

Considering the improvement of neuroinflammation, we were interested in the effect of DI administration on cognitive impairment induced by *T. gondii* infection. In the novel location test, in the case total exploration time was not significantly different between the two groups (*P* > 0.05, **Figure 5B**), DI significantly improved place recognition memory with increasing the place discrimination index (percentage of time spent with the object in a novel place) in mice compared with Tg+Veh mice (*P* < 0.05, **Figures 5A, C**). In the novel object recognition test, the novel object discrimination index (percentage of time spent with novel object) was significantly decreased in Tg+Veh group compared with the control and Tg+DI group (*P* < 0.05, **Figures 5D, F**), although the total exploration was not significantly different among the four groups (*P* > 0.05, **Figure 5E**). In the Y-maze spatial memory test, the Tg + DI group spent more time in exploring the novel arm than that of the Tg+Veh mice (*P* < 0.05, **Figures 5G, H**). In the nest building test, the Tg + DI group had higher deacon nest score (ability to build a nest) than that of the Tg+Veh mice (*P* < 0.01) without a significant difference to control mice (*P* > 0.05, **Figures 5I, K**). In contrast, the untorn nestlet weight (nest-building deficit) of Tg+DI groups was significantly decreased compared with that of the Tg+Veh group (*P* < 0.05, **Figure 5J**). However, the cognition index of the Tg+DI group in nest building test did not return to the level of the control group. Therefore, DI attenuated the impairment of cognitive function induced by *T. gondii* chronic infection.

### 3.6 Dimethyl itaconate alleviates synaptic impairment in *Toxoplasma gondii* infected mice

**Figure 6:**
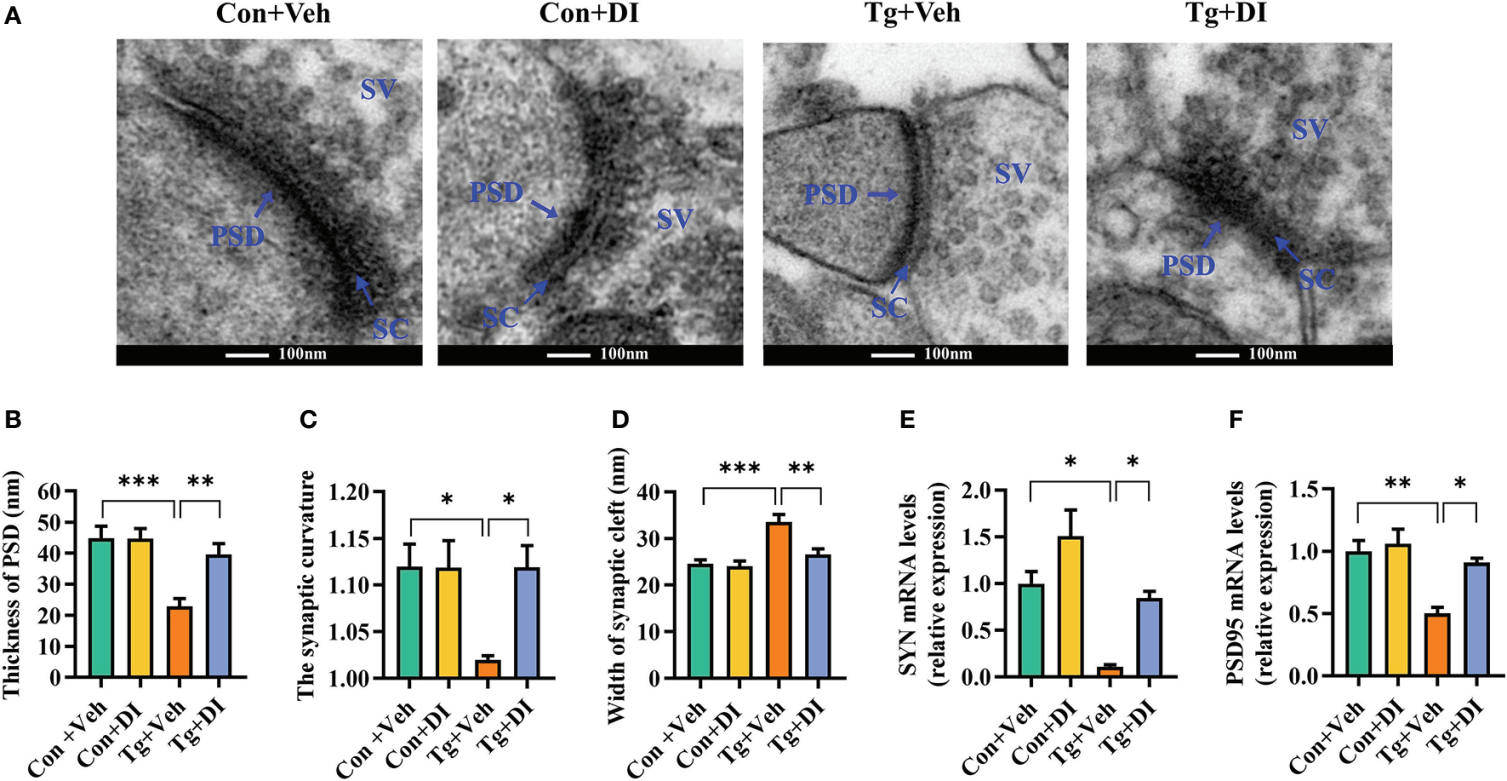
Dimethyl itaconate alleviates synaptic impairment in *T. gondii* infected mice. **(A)** The ultrastructure of synapses in the hippocampus CA1 region of mice on the electron micrograph (scale bar 100 nm). **(B–D)** Image analysis of the thickness of postsynaptic density (PSD), the synaptic curvature, and width of the synaptic cleft (SC) (*n* = 2, 10 images per mouse). **(E, F)** The mRNA expression of SYN and PSD95 in the hippocampus (*n* = 3). Con+Veh, control mice with vehicle control treatment; Con+DI, control mice with DI treatment; Tg+Veh, *T. gondii* infected mice with vehicle control treatment; Tg+DI: *T. gondii* infected mice with DI treatment. Values are mean ± SEM. *P<0.05, **P<0.01, ***P<0.001.

Integrity of synaptic ultrastructure and plasticity is required for cognitive function (65). Following our finding that DI improves the cognitive decline, we further evaluated the profile of synaptic ultrastructure in the CA1 region of hippocampus. Using transmission electron microscopy, we observed that Tg mice showed decreased thickness of the postsynaptic densities (PSD), broadened synaptic cleft (SC) and lower synaptic curvature of the pyramidal neurons in hippocampal CA1 region compared to Con mice (*P* < 0.05, **Figures 6A-D**). However, compared with Tg+Veh group, DI significantly improved these synaptic ultrastructure impairments, exhibiting thicker PSD, narrower SC and higher synaptic curvature (*P* < 0.05, **Figures 6A-D**). Moreover, we showed that the mRNA levels of pre- and post-synaptic proteins (SYN and PSD95) were significantly downregulated in the hippocampus of infected mice, which was restored after DI administration (*P* < 0.05, **Figures 6E, F**). Overall, these results indicated that DI can alleviate the impairment of synaptic ultrastructure and the deficits of synaptic plasticity-associated genes in *T. gondii-*infected mice, thereby contributing to the improvement of cognition.

### 3.7 Dimethyl itaconate exerts a therapeutic potential on *Toxoplasma gondii*-induced cognitive and synaptic impairments

**Figure 7:**
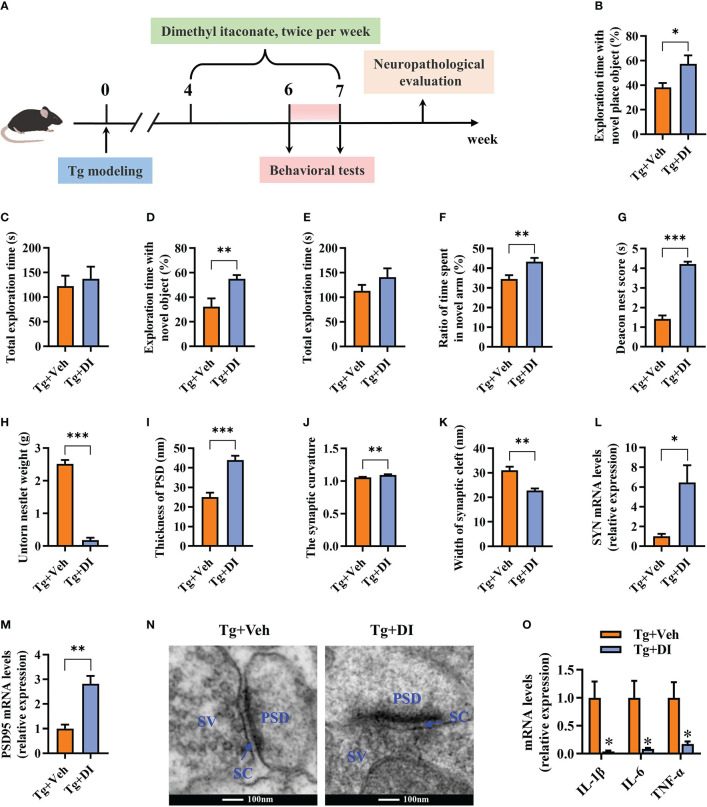
Dimethyl itaconate has the therapeutic effect on *T. gondii*-induced cognitive and synaptic impairments in mice. **(A)** Schematic timeline for DI therapeutic effect on cognitive deficits induced by *T. gondii* infection in mice. **(B)** Percentage of time spent with the object in the novel place to total object exploration time and **(C)** the total object exploration time in the novel location test were recorded. **(D)** Percentage of time spent with the novel object to total object exploration time and **(E)** the total object exploration time in the novel object recognition test were recorded. **(F)** Percentage of time spent with the novel arm to total arm exploration time in Y-maze spatial memory test. **(G)** The nest score and **(H)** untorn nestlet weight (amount of untorn nesting material) (*n* = 10 mice for each group). **(I–K)** Image analysis of the thickness of postsynaptic density (PSD), the synaptic curvature, and width of the synaptic cleft (SC) (*n* = 2, 10 images per mouse). **(L, M)** The mRNA expression of SYN and PSD95 in the hippocampus (*n* = 3). **(N)** The ultrastructure of synapses in the hippocampus CA1 region of mice in Tg+Veh group compared with Tg+DI group on the electron micrograph (scale bar 100 nm). **(O)** The mRNA expression of IL-1β, IL-6 and TNF-α in the hippocampus (*n* = 3). Tg+Veh, *T. gondii* infected mice with vehicle control treatment; Tg+DI: *T. gondii* infected mice with DI treatment. Values are mean ± SEM. *P<0.05, **P<0.01, ***P<0.001.

To explore the potential therapeutic effect of DI on the cognitive impairment induced by *T. gondii*, DI were intraperitoneally administrated in C57BL/6J mice after 4 weeks of *T. gondii* infection (**Figure 7A**). In comparison with Tg+Veh group, Tg+DI group showed higher cognitive index, manifesting a higher percentage of time spent with the object in a novel place in novel location test (*P* < 0.05, **Figures 7B, C**), higher percentage of time spent with new object in novel object test (*P* < 0.01, **Figures 7D, E**), increasing the novel place discrimination index in Y-maze spatial memory test (*P* < 0.01, **Figure 7F**), and a better nesting building ability in nest building test (*P* < 0.001, **Figures 7G, H**). Notably, the difference of behavior tests was not due to the variant general activity because the two groups had similar total exploration time and distance during the test phase (*P*> 0.05, **Figures 7C, E**; **Supplementary Figures 4A, B**). Furthermore, DI administration increased the body weight gain (*P*> 0.05, **Supplementary Figure 4C**), and reduced the piloerection phenomenon in *T. gondii* infected mice. Compared with Tg+Veh group, the mice in Tg+DI group exhibited thicker postsynaptic densities, higher synaptic curvature and narrower synaptic cleft of the pyramidal neurons in the hippocampal CA1 region (*P* < 0.01, **Figures 7I–K, N**). In consistent with the results of the transmission electron microscopy, we found that the mRNA levels of SYN and PSD95 were upregulated in the hippocampus of infected mice post DI administration (*P* < 0.05, **Figures 7L, M**). In addition, DI could significantly downregulate the mRNA expression of pro-inflammatory cytokines (*P* < 0.05, **Figure 7O**). Taken together, these results demonstrated that DI can treat cognitive and synaptic impairments induced by *T. gondii* infection.

## 4 Discussion

**Figure 8:**
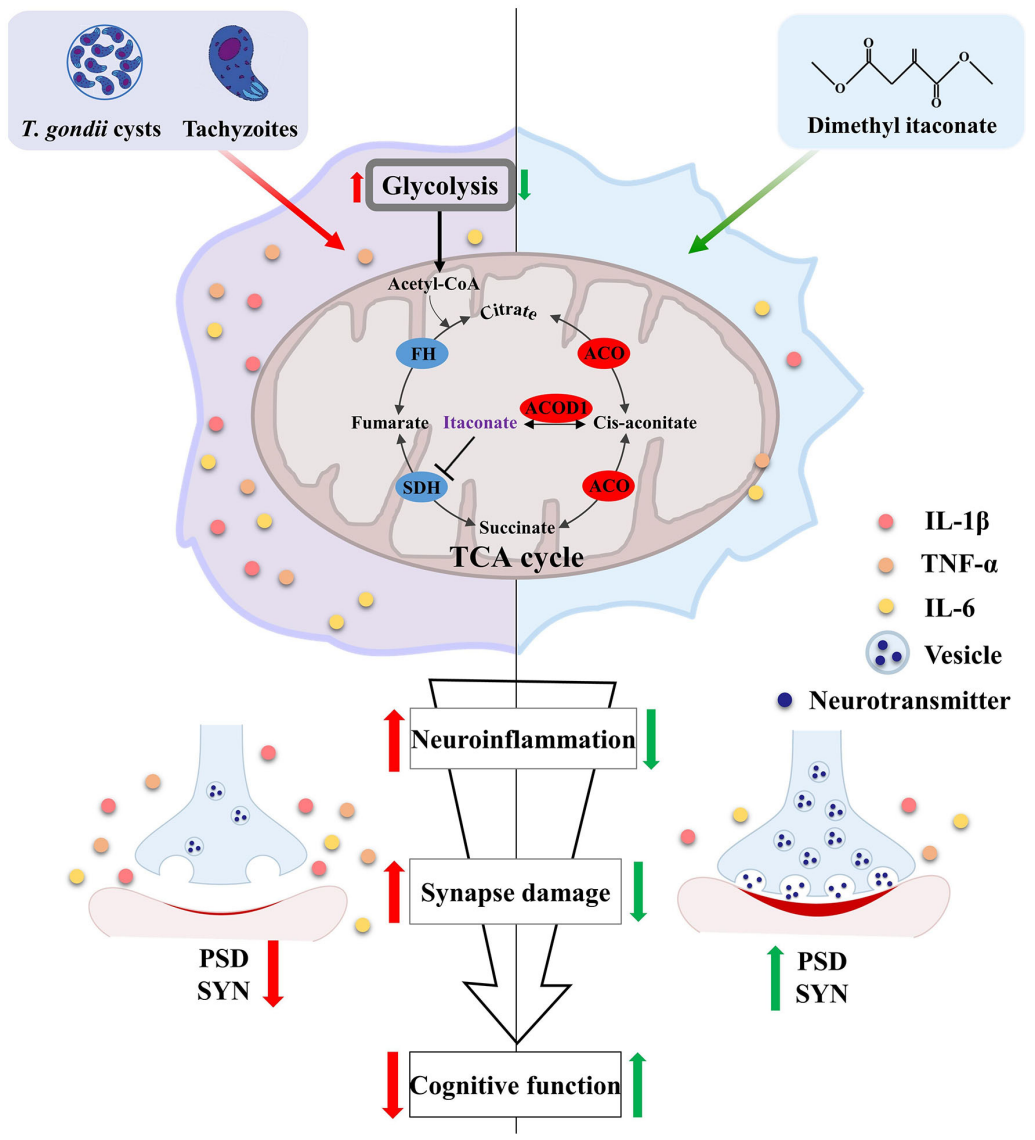
Schematic strategy of how dimethyl itaconate improves the cognitive deficits induced by *T. gondii* infection. *T. gondii* infection induces neuroinflammation along with Acod1/itaconate axis disorder in the hippocampus of mice. These activated immune cells (*e. g.* microglia) can impair the synaptic plasticity *via* engulfing the synaptic plasticity-related proteins (SYN and PSD95), thereby causing cognitive deficits. However, DI supplementation can significantly attenuate the cognitive deficits and neuropathological changes induced by *T. gondii* infection. Red arrows represent the consequent events following *T. gondii* infection; Green arrows represent DI’s pro-cognitive effects.

The evidence in recent years implicates that chronic infection of *T. gondii* is closely associated with neurodegenerative diseases including AD (11, 66). Thus, it is urge to uncover the underlying mechanism of how the parasite impairs cognitive function. In the present study, using TgCtwh6 chronically infected mice model, we demystified that *T. gondii* induced cognitive deficits accompanied by impairment of synaptic ultrastructure and plasticity. In support, the parasitic infection significantly silenced the expression of key genes associated with cognition, behavior, and synaptic plasticity in the hippocampus. Moreover, the infection induced extensive hippocampal inflammatory responses, which were characterized by the activation of microglia. Interestingly, the disorder of Acod1/itaconate axis, a critical node that masters immunity and metabolism (67), was identified post infection. We unveiled that supplementation of the itaconate derivative (DI) effectively attenuated cognitive deficits induced by the parasitic infection *via* relieving neuroinflammation and impairment of synaptic ultrastructure and plasticity (**Figure 8**). Overall, these findings provide a novel insight for the pathogenesis of *T. gondii*-related cognitive deficits in hosts, and also lay a basis for the application of DI in preventing neurodegenerative diseases.

The hippocampus is responsible for cognitive processing, spatial learning and memory (12–14). The dysfunction of hippocampus has been indicated in the models of AD (15–17). The present study showed that Tg chronic infection has a tremendous impact on the global expression of genes in the hippocampus of mice, which indicated that the neuropathological changes in hippocampus may be the basis for *T. gondii*-induced cognitive deficits. In fact, different brain areas manipulate differential behaviors. For example, the prefrontal cortex is a region that contributes to cognition controlling abilities *via* providing structural basis for complex goal-directed behavior (68); the amygdala is a region responsible for social interaction, the formation/consolidation of social recognition memory (9, 69), and orchestrating a switch in fear state (25). It is reported that *T. gondii* infection can also induce the behavior alteration that related to the two brain regions in mice (9, 25, 68). Thus, future investigation should focus on multiple brain regions.

The reduction of synaptic plasticity is the basis for the onset and progression of cognitive impairment and memory deficits in AD (70, 71). Using transmission electron microscopy, we found a thinner postsynaptic density, a wider synaptic cleft and lower synaptic curvature in the hippocampus of infected mice, suggesting the impaired synaptic plasticity in the hippocampus. In line with this, our transcriptome results showed that the down-regulated differential genes were significantly enriched into biological processes related to synaptic plasticity and synaptic transmission. SYN and PSD95 are critical for structural plasticity and functional integrity of synapses, and consequently learning and memory cognitive function (72, 73). This study found that the two genes were significantly downregulated in the hippocampus of infected mice, which was also observed in a previous study (31). In addition, several studies reported the neuron impairments such as the reduced dendritic spine length and density post *T. gondii* infection (30, 31). Therefore, the impairment of neuron integrity might be a vital event in the parasite-induced cognitive deficits. GABA, as a main inhibitory neurotransmitter in the CNS, participated in a wide range of physiological processes concerning the regulation of cognition, learning, memory and neural development (74). In addition, GABA engages in cortico-hippocampal information processing, which plays a critical role in spatial navigation and contextual memory (75). Dysfunction in GABAergic signaling is known to be a central factor in the pathogenesis of several neurological disorders (76, 77). In the present study, GSEA analysis showed that many downregulated genes were mapped in the GABAergic system post infection. Another study also reported a disorder of GABA transmission in the hippocampus of *T. gondii* infected mice (78). Collectively, these results indicated that the impaired synaptic plasticity and transmission, may contribute to the cognitive decline induced by *T. gondii* infection.

Neuroinflammation, characterized by the activation of microglia and astrocytes, plays a vital role in the progression of neurodegenerative diseases (19, 79, 80). We observed that *T. gondii* infection dramatically increased the number of Iba1^+^ microglia and upregulated the proinflammatory cytokines. Moreover, the transcriptome analysis of hippocampus showed massive DEGs were enriched in the inflammatory pathways. Notably, we found that the expression of specific markers of M1 type microglia (Aif1, Cd86, Cd40, Ccl2, Ccr2, Cx3cr1, IL-1β, IL-6, and NOS2) (60, 61) was elevated post infection. It is well-known that microglia constantly detect signs of pathogenic invasion or tissue damage, thereby maintaining brain homeostasis (81). M1 type microglia can secrete inflammatory cytokines and chemokines (81, 82). Zhao et al. found that these cytokines can directly induce neuronal apoptosis and synaptic dysfunction (22), subsequently resulting in cognitive impairment. Furthermore, microglia can also induce synaptic damage and neuronal degeneration *via* complement system-mediated synaptic engulfing (23, 83). We observed the upregulated transcripts of complement family members post *T. gondii* infection, which was in accordance with the impaired synaptic ultrastructure in the infected mice. Some other studies also demonstrated that *T. gondii* infection induces M1-type microglia polarization *in vitro* (84) and *in vivo* (85). It is most likely that microglia might be attributable to the neuronal injure and synaptic loss, jointly resulting in the cognitive deficits induced by *T. gondii*. Indeed, multiple studies have recognized that the neuroinflammation may be one crucial factor that potentially contributes to behavioral changes induced by the parasite (25–29).Thus, it is possible that alleviating neuroinflammation may improve the parasite-induced cognitive impairment.

The emerging discipline immunometabolism opens the road to manipulate neuroinflammation *via* metabolic reprogramming (32, 33). In the present study, we firstly characterized the metabolic phenotype in the hippocampus post *T. gondii* infection. We found the Acod1 and its related pathways (including Mdh2, Idh2, A20, Hmox1, Nfe2l2, Tap1 and Atf3) in the TCA were significantly disturbed in the hippocampus of mice post *T. gondii* infection. Notably, such expression profile of these genes was also observed in the cerebral cortexes of mice chronically infecting with the tachyzoites of *T. gondii* (86). Moreover, the Acod1 is upregulated in the whole brain of mice infecting with the oocysts of *T. gondii* (87). These results including ours jointly indicate that dysfunction of Acod1 may be a common event in the brain post *T. gondii* infection. Interestingly, *T. gondii* priming and rechallenging is reported to elevate the expression of Acod1 in the hippocampal microglia of mice (88). Thus, it is speculated that Acod1 may manipulate the neuroinflammation *via* regulating the metabolic shift in microglia.

Itaconate, the metabolite synthesized by the enzyme encoded by Acod1, has attracted extensive interests due to its immunomodulatory activity in LPS-stimulated macrophages (41). Notably, a recent study has shown that the derivative of itaconate suppresses aerobic glycolysis to exert intense anti-inflammatory effect in macrophages (38). These findings attracted us to test if itaconate could protect the cognitive impairment *via* alleviating neuroinflammation in *T. gondii* infected mice. Here, we demonstrated that DI, one derivative of itaconate, is a potential drug candidate against *T. gondii*-induced cognitive deficits. DI supplementation significantly prevented cognitive impairment, evidenced by the behavior performance, amelioration of neuroinflammation and improved synaptic plasticity or ultrastructure in the hippocampus. In detail, DI supplementation effectively improved cognitive decline in novel location, novel object recognition test, Y-maze spatial memory and nesting building test induced by *T. gondii*, indicating enhanced cognitive processing, spatial memory and ability to perform activities of daily living (52–54, 56). Using transmission electron microscope, we observed a thicker postsynaptic density, a narrower synaptic cleft and increased synaptic curvature in the hippocampus of infected mice after DI supplementation, which showed improvement of synaptic impairment induced by infection. Furthermore, DI supplementation could alleviate microglia activation induced by *T. gondii*, thus improving the parasite-induced cognitive impairment.

Interestingly, our data also indicated the therapeutic potential of DI in *T. gondii*-induced cognitive decline. After 4 weeks post infection, the mice showed impaired cognitive function. However, successive DI administration for 2 weeks could significantly ameliorate the cognitive deficits. Neurodegenerative diseases including AD is currently one of unsolvable problems worldwide due to the complex pathogenesis of the disease and lack of effective intervention strategies (2, 89). Our data provide a clue that DI may be disease-modifying drug for *T. gondii*-related neurodegenerative diseases. Notably, two recent studies supported that DI can alleviate neuroinflammation (46), and attenuate memory impairment in the mice model of AD (47). In future, clinical trials and mechanistic investigations should be further performed to demonstrate the therapeutic value of DI in neurodegenerative diseases including *T. gondii*-related cognitive deficits.

## 5 Conclusion

Overall, the present study demonstrated that *T. gondii* chronic infection induced cognitive deficits, accompanied by neuroinflammation and impaired synaptic ultrastructure. Hippocampal transcriptome showed that the infection suppressed the expression of key genes associated with synapse plasticity, transmission, behavior and synaptic excitability while upregulating proinflammatory profiles characterized by microglial activation. Interestingly, the disorder of Acod1-itaconate axis were identified to be the metabolic phenotype in the hippocampus post infection. Notably, we provided the evidence that DI could attenuate the cognitive deficits induced by *T. gondii* infection *via* improvement of synaptic ultrastructure and neuroinflammation. Overall, these findings provide a novel insight for the pathogenesis and the intervention of *T. gondii*-related cognitive deficits in hosts.

## Data availability statement

The datasets presented in this study can be found in online repositories. The names of the repository/repositories and accession number(s) can be found below: https://www.ncbi.nlm.nih.gov/sra; PRJNA859430.

## Ethics statement

The animal study was reviewed and approved by Ethics Committee of Xuzhou Medical University.

## Author contributions

WP, YY, XY and KZ designed the research study and revised the manuscript. YH, DX and ZY performed the research, analyzed the data, and wrote the manuscript. YW, YZ, XT, JZ, ZL and WC analyzed the data. All authors contributed to the article and approved the submitted version.

## Glossary

**Table:**

DI	dimethyl itaconate
Acod1	aconitate decarboxylase 1
AD	Alzheimer’s disease
Tg	*Toxoplasma gondii*
Con	control
Veh	vehicle
TEM	transmission electron microscope
IF	immunofluorescence
SYN	synaptophysin
PSD95	postsynaptic density 95
SC	synaptic cleft
SV	synaptic vesicle
Iba1	ionized calcium-binding adaptor molecule1
IL-1β	interleukin-1 beta
IL-6	interleukin-6
TNF-α	tumor necrosis factor-α
IRG1	immune responsive gene 1
TCA	tricarboxylic acid
RNA-seq	genome-wide RNA-sequencing
GSEA	gene set enrichment analysis
NES	normalized enrichment score
SEM	standard error of the mean
ANOVA	analysis of variance
FDR	false discovery rate
NL	novel location
NOR	novel object recognition
ddH2O	double-distilled water
CA1	cornu ammonis 1
CA3	cornu ammonis 3
DG	dentate gyrus
PBS	phosphate buffered solution
qPCR	quantitative real-time PCR
DEGs	differential expression genes
GO	Gene Ontology
KEGG	Kyoto Encyclopedia of Genes and Genomes
P value	probability
CC	cellular component
MF	molecular function
BP	biological process
GABA	γ-aminobutyric acid
NF-κB	nuclear factor kappa-B
JAK-STAT	Janus kinase-signal transducer and activator of transcription
Aif1	allograft inflammatory factor 1
Cd86	cluster of differentiation 86
Cd40	cluster of differentiation 40
Ccl2	C-C motif ligand 2
Ccr2	C-C motif chemokine receptor 2
Cx3cr1	C-X3-C motif chemokine receptor 1
Nos2	nitric oxide synthase 2
Sdhaf3	succinate dehydrogenase assembly factor 3
Sucla2	succinate-CoA ligase [ADP-forming] subunit beta
Suclg2	succinate–CoA ligase [GDP-forming] subunit beta
Idh3a	isocitrate dehydrogenase 3 catalytic subunit alpha
Mdh2	malate dehydrogenase 2, mitochondrial
Atf3	activating transcription factor 3
Nqo2	N-ribosyldihydronicotinamide: quinone reductase 2
Stat3	signal transducer and activator of transcription 3
Psmb9	proteasome 20S subunit beta 9
Nfe2l2	nuclear factor erythroid-derived 2 like 2
Nrf2	nuclear factor erythroid2-related factor 2
Hk	hexokinase
Phi	phosphate isomerase
Pfkfb	fructose-2,6-biphosphatase
Pfk	phosphofructokinase
Aldo	aldolase
Gapdh	glyceraldehyde-3-phosphate dehydrogenase
Pgk1	phosphoglycerate kinase 1
Pk	pyruvate kinase
Pdh	pyruvate dehydrogenase complex
Cs	citrate synthase
Aco	aconitase
Idh	isocitrate dehydrogenase
αKGDH	α-ketoglutarate dehydrogenase
Sdh	succinate dehydrogenase
Fh	fumarate hydratase
Mdh	malate dehydrogenase
CNS	central nervous system
Tnfaip3 (A20)	tumor necrosis factor alpha-induced protein 3
Hmox1	heme oxygenase 1
Tap1	transporter 1
LPS	lipopolysaccharide
