# Supplementary material for: A metabolite attenuates neuroinflammation, synaptic loss and cognitive deficits induced by chronic infection of Toxoplasma gondii
Source: Front Immunol. 2022 Dec 22;13:1043572. doi: 10.3389/fimmu.2022.1043572 (PMC9815861; doi:10.3389/fimmu.2022.1043572)
Supplement: Supplementary file 1 [file DataSheet_1.docx]

**A metabolite attenuates neuroinflammation, synaptic loss and cognitive deficits induced by chronic infection of *Toxoplasma gondii***

**Yan He^1,2,3†^, Daxiang Xu^1†^, Ziyi Yan^1,2,3†^, Yongshuai Wu^1,2,3^, Yongsheng Zhang^1,2,3^, Xiaokang Tian^1,2,3^, Jinhang Zhu^1,3,4^, Zhuanzhuan Liu^1^, Wanpeng Cheng^1^, Kuiyang Zheng^1^, Xiaoying Yang^1*^, Yinghua Yu^1*^, Wei Pan^1*^**

^1^ Jiangsu Key Laboratory of Immunity and Metabolism, Department of Pathogen Biology and Immunology, Xuzhou Medical University, Xuzhou, Jiangsu, China

^2^ The First Clinical Medical College, Xuzhou Medical University, Xuzhou, Jiangsu, China

^3^ National Experimental Teaching Demonstration Center of Basic Medicine (Xuzhou Medical University), Xuzhou, Jiangsu, China

^4^ The Second Clinical Medical College, Xuzhou Medical University, Xuzhou, Jiangsu, China

**^†^These authors share first authorship on this work.**

^*^**Correspondence**:

panwei525@126.com (Wei Pan); yinghua@uow.edu.au (Yinghua Yu); yxyxiaoliqq@163.com (Xiaoying Yang)

**Supplementary Information**

**
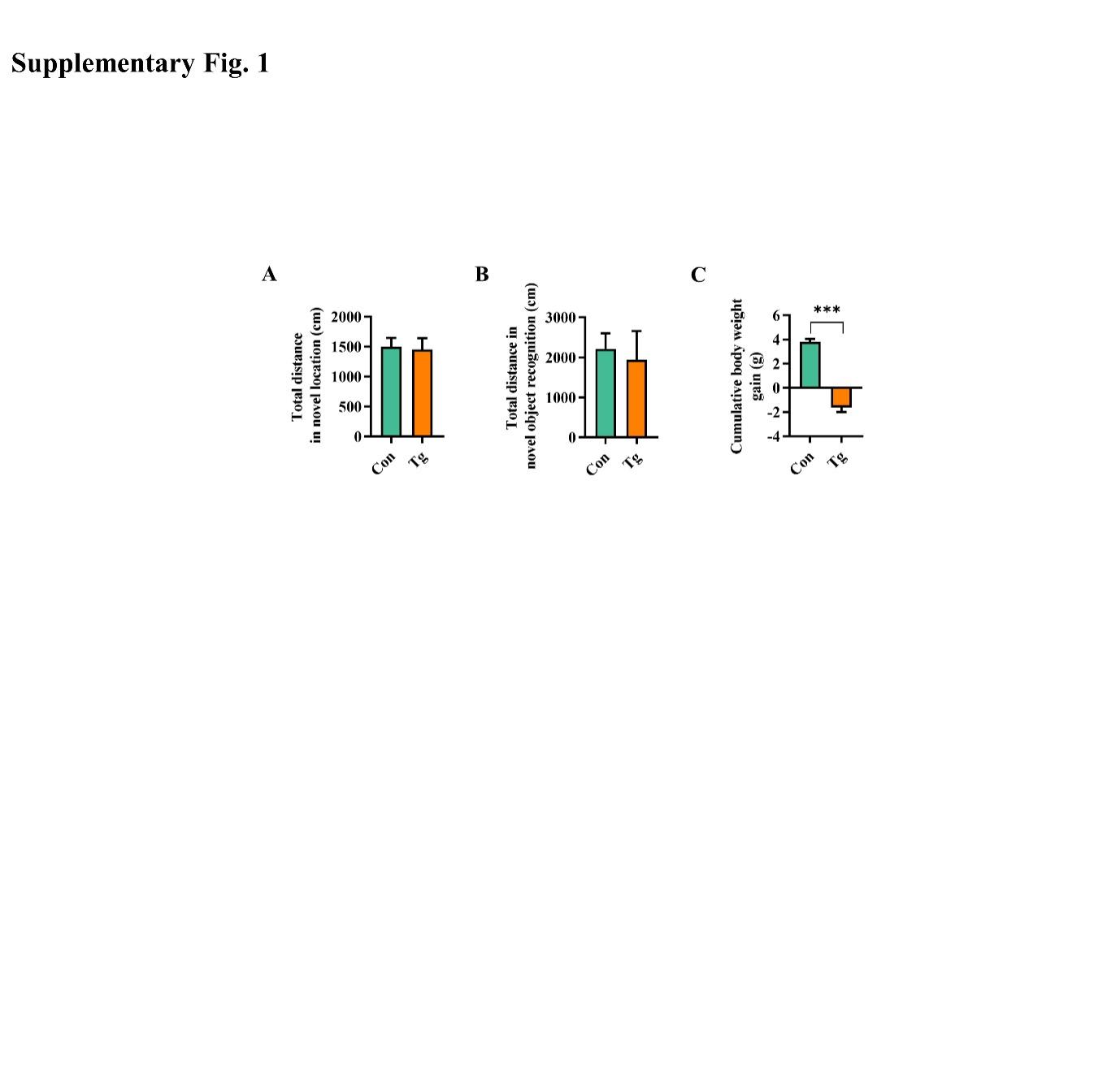
**

**Supplementary Figure 1.** **The effects of *T. gondii* chronic infection on normal health parameters in mice.**

The mice were infected with 10 cysts of *T. gondii*. The normal health parameters were monitored at the 7^th^ week. **A** The total distance in the novel location test (*n* = 10). **B** The total distance in the novel object recognition test (*n* = 10). **C** The cumulative body weight gain (*n* = 4-5). Con, vehicle control mice; Tg, *T. gondii* infected mice. Values are mean ± SEM. ****P*<0.001.

**
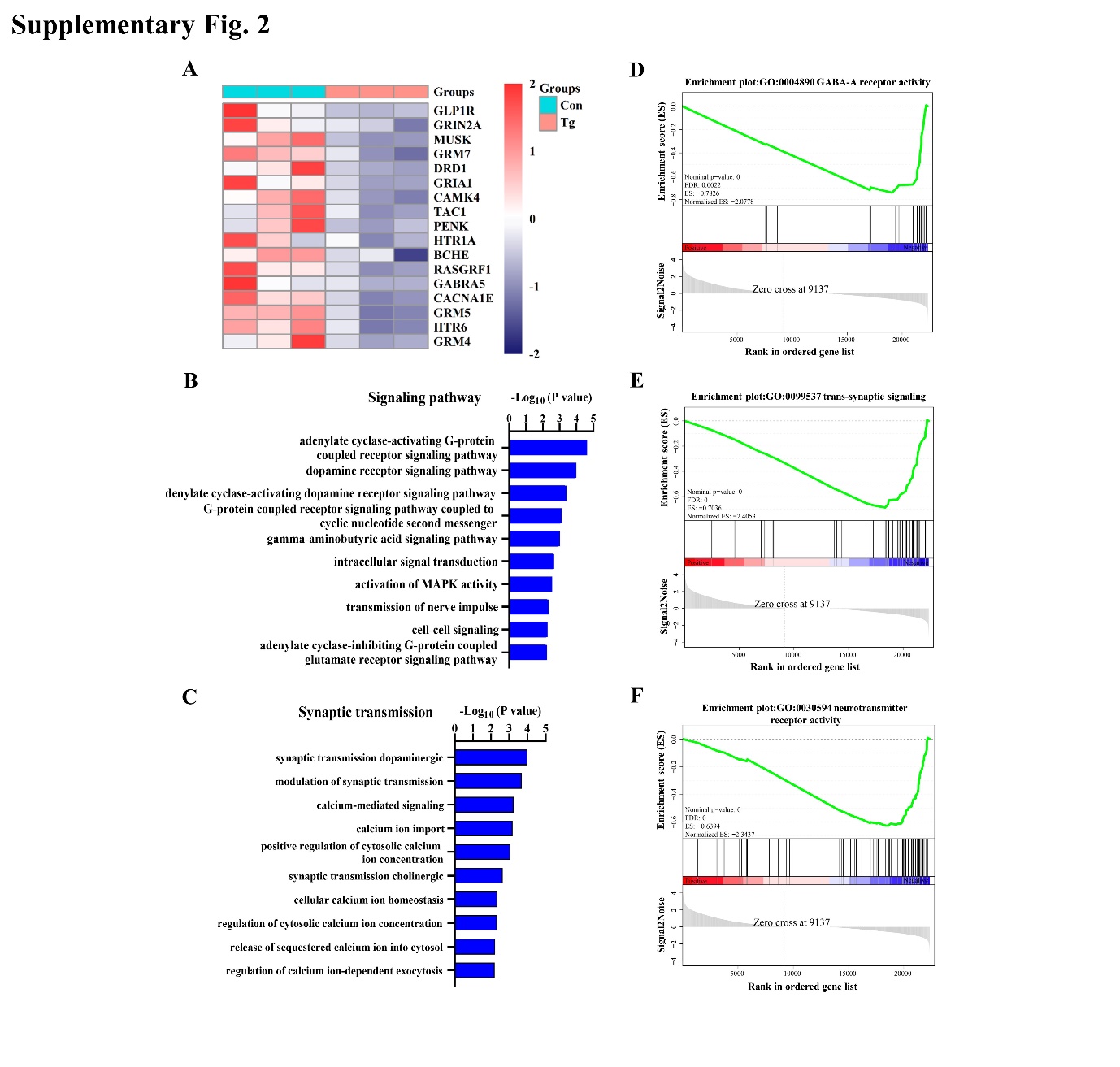
**

**Supplementary Figure 2. RNA sequencing analysis reveals the dysregulated synaptic transmission in the hippocampus of *T. gondii*-infected mice.**

**A** The downregulated expression of core genes related to behavior post infection. **B** The biological processes associated with signaling pathway post infection. **C** The biological processes associated with synaptic transmission. Enrichment plots of (**D**) GABA-A receptor activity, (**E**) trans-synaptic signaling, and (**F**) neurotransmitter receptor activity in GSEA analysis. *n* = 3 mice for each group. Con, vehicle control mice; Tg, *T. gondii* infected mice.

**
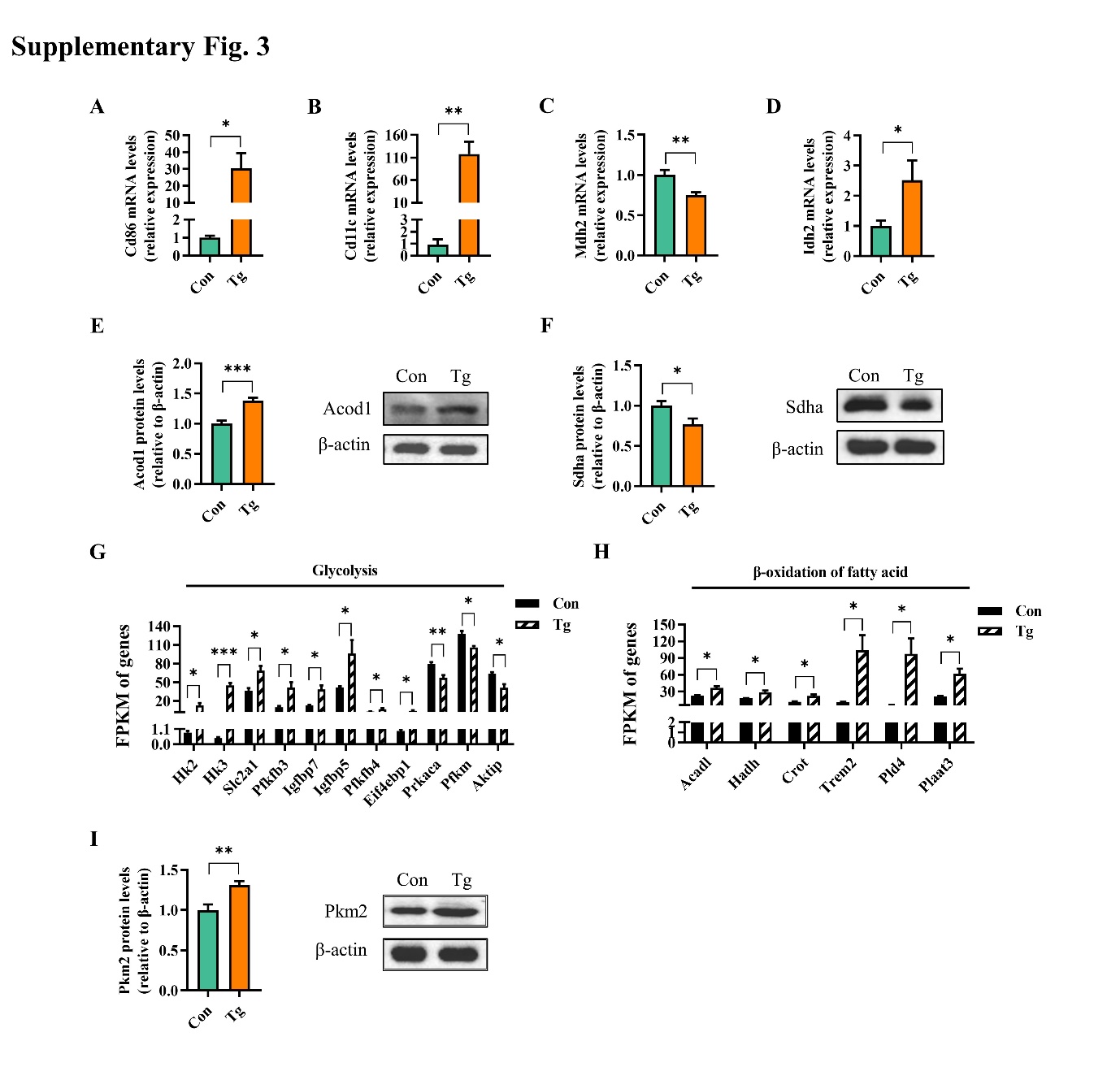
**

**Supplementary Figure 3. Validation of RNA sequencing in the hippocampus of mice chronic infection with *T. gondii*.**

The mRNA expression levels of Cd86 (**A**), Cd11c (**B**), Mdh2 (**C**) and Idh2 (**D**) were detected in the hippocampus of mice post infection (*n* = 4-6). The protein levels of Acod1 (**E**) and Sdha (**F**) were determined in the hippocampus of mice post infection (*n* = 6). Normalized expression of selected genes regulating glycolysis (**G**) and fatty acid β-oxidation (**H**) (*n* = 3). The protein levels of Pkm2 (**I**) were determined in the hippocampus of mice post infection (*n* = 6). Con, vehicle control mice; Tg, *T. gondii* infected mice. Values are mean ± SEM. ^*^*P*<0.05, ^**^*P*<0.01, ^***^*P*<0.001.


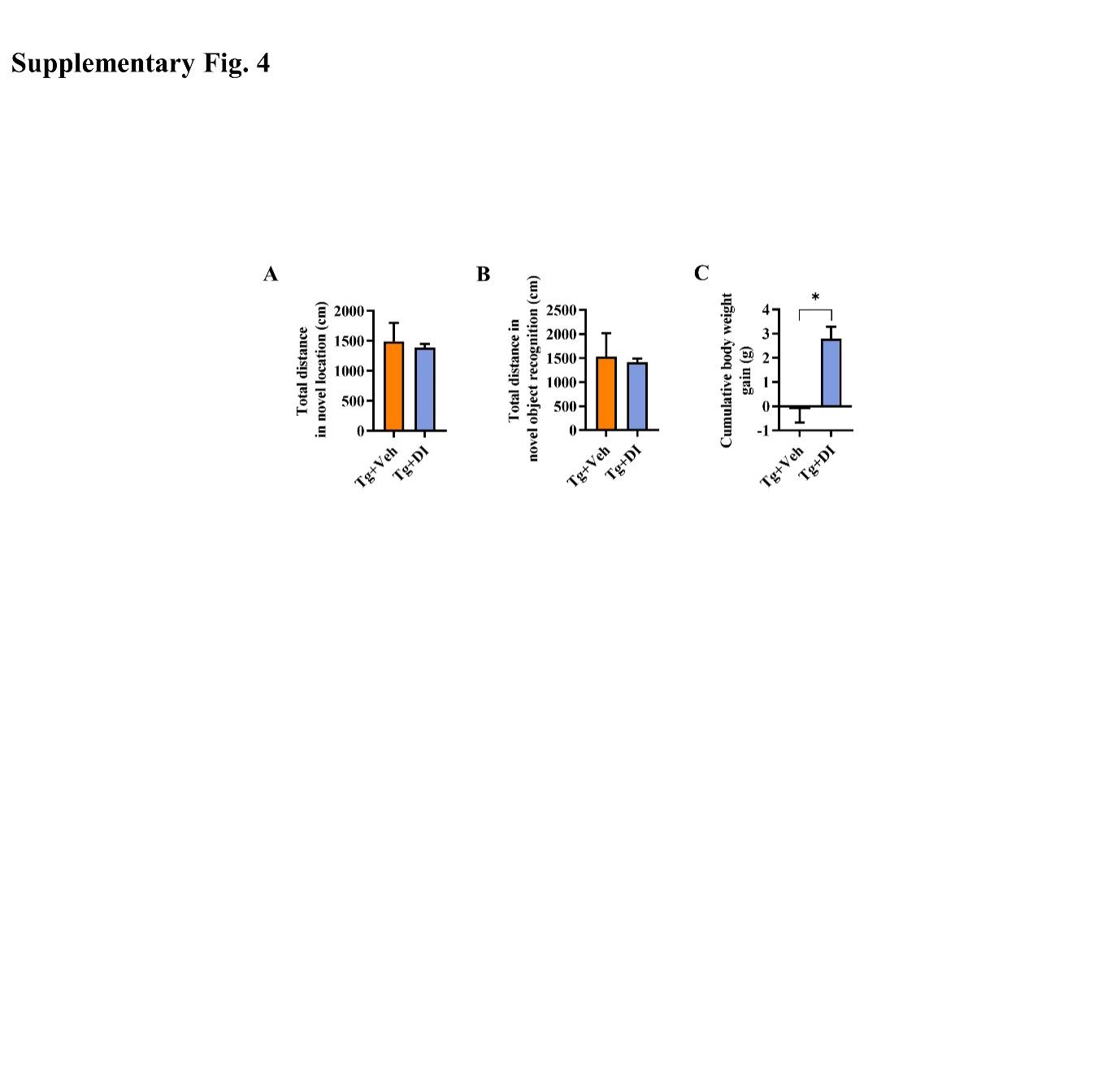


**Supplementary Figure 4.** **The effects of DI treatment on normal parameters in mice.**

The mice were infected with 10 cysts of *T. gondii* for 4 weeks, followed by successive DI administration for 2 weeks. The normal health parameters were monitored at the 7^th^ week. **A** The total distance in the novel location test (*n* = 10). **B** The total distance in the novel object recognition test (*n* = 10). **C** The cumulative body weight gain (*n* = 4-5). Tg+Veh, *T. gondii* infected mice with vehicle control treatment; Tg+DI: *T. gondii* infected mice with DI treatment. Values are mean ± SEM. ^*^*P*<0.05.

**Supplementary Table 1 The qRT-PCR primer sequences used in this study**

| NO. | Gene Symbol | Forward primer (5’-3’) | Reverse primer (5’-3’) |
| --- | --- | --- | --- |
| **1** | β-actin | AGAAGGTGGTGAAGCAGGCATC | CGAAGGTGGAAGAGTGGGAGTTG |
| **2** | SYN | CGCACCTCGGACAAGTCTC | CCCGAAGGCGAAAATAGCAAA |
| **3** | PSD95 | TCCGGGAGGTGACCCATTC | TTTCCGGCGCATGACGTAG |
| **4** | IL-1β | TGGGAAACAACAGTGGTCAGG | CTGCTCATTCACGAAAAGGGA |
| **5** | IL-6 | TCACAGAAGGAGTGGCTAAGGACC | ACGCACTAGGTTTGCCGAGTAGAT |
| **6** | TNF-α | CTTGTTGCCTCCTCTTTTGCTTA | CTTTATTTCTCTCAATGACCCGTAG |
| **7** | CD86 | GTCTAAGCAAGGTCACCCGAAAC | TCCAGAACACACACAACGGTCATA |
| **8** | CD11c | CTGGATAGCCTTTCTTCTGCTG | GCACACTGTGTCCGAACTCA |
| **9** | MDH2 | ATTTTGTGGTAGATCGAGCTGG | CCTCCGGCAGGGAAGTTATAC |
| **10** | IDH2 | AAGGCTACCTTGGACCGGAG | CATCACAACCTTTGAGGCAATCT |
